# Supplementary material for: The interactome of intact mitochondria by cross-linking mass spectrometry provides evidence for coexisting respiratory supercomplexes
Source: Mol Cell Proteomics. 2017 Dec 8;17(2):216–32. doi: 10.1074/mcp.RA117.000470 (PMC5795388; doi:10.1074/mcp.RA117.000470)
Supplement: Supplemental Data [file supp_RA117.000470_133922_0_supp_23978_fzffwf.zip › spectra_annotation/mito_spectra_annotation/171-1-4-1-9-1.pdf]

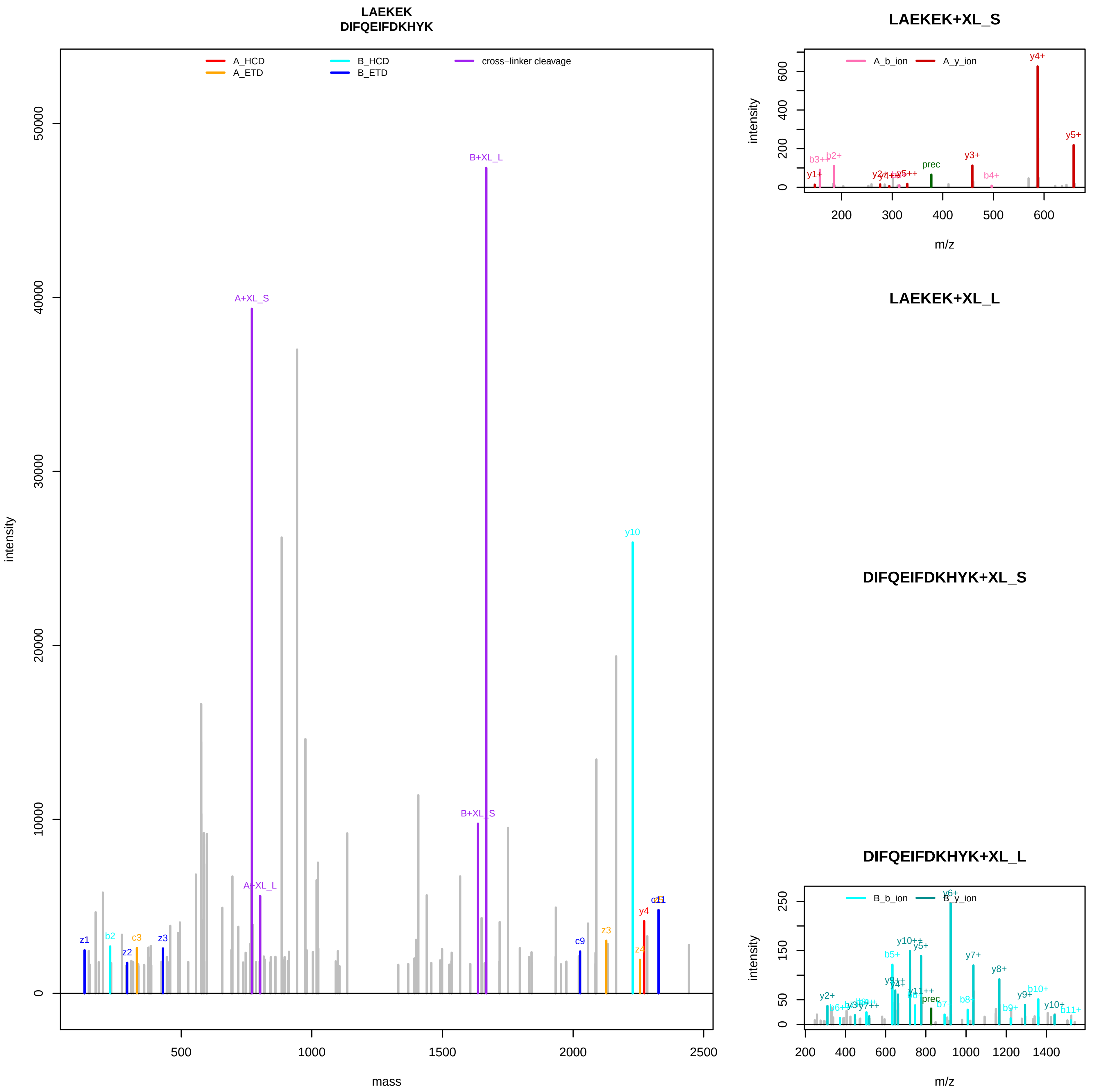

LAEKEK
DIFQEIFDKHYK
A_HCD
A_ETD
B_HCD
B_ETD
cross−linker cleavage
B+XL_L
A+XL_S
A+XL_L
B+XL_S
y10
y4
c11
z3
z4
c9
z1
b2
z2
c3
z3
intensity
mass
0
10000
20000
30000
40000
50000
500
1000
1500
2000
2500
LAEKEK+XL_S
A_b_ion
A_y_ion
y4+
y5+
y3+
prec
b4+
b3++
b2+
y1+
y2+
y5++
intensity
m/z
0
200
400
600
200
300
400
500
600
LAEKEK+XL_L
DIFQEIFDKHYK+XL_S
DIFQEIFDKHYK+XL_L
B_b_ion
B_y_ion
y6+
y10++
b5+
y5+
y7+
y8+
y9++
y4+
y11++
prec
y2+
b6+
y3+
y7++
b7+
b8+
b9+
y9+
b10+
y10+
b11+
intensity
m/z
0
50
150
250
200
400
600
800
1000
1200
1400
